# Microbial Diversity Characteristics of Areca Palm Rhizosphere Soil at Different Growth Stages

**DOI:** 10.3390/plants10122706

**Published:** 2021-12-09

**Authors:** Siyuan Ma, Yubin Lin, Yongqiang Qin, Xiaoping Diao, Peng Li

**Affiliations:** 1 Ministry of Education Key Laboratory for Ecology of Tropical Islands, Hainan Provincial Key Laboratory for Tropical Plant and Animal Ecology, College of Life Sciences, Hainan Normal University, Haikou 571158, China; 2018207130001@hainnu.edu.cn (S.M.); 20202071300171@hainnu.edu.cn (Y.Q.); 2 State Key Laboratory of Marine Resource Utilization in South China Sea, College of Ecology and Environment, Hainan University, Haikou 570228, China; 19071300210013@hainanu.edu.cn

**Keywords:** *Areca catechu*, microbiome, interaction network, plant development, high-throughput sequencing

## Abstract

The rhizosphere microflora are key determinants that contribute to plant health and productivity, which can support plant nutrition and resistance to biotic and abiotic stressors. However, limited research is conducted on the areca palm rhizosphere microbiota. To further study the effect of the areca palm’s developmental stages on the rhizosphere microbiota, the rhizosphere microbiota of areca palm (*Areca catechu*) grown in its main producing area were examined in Wanning, Hainan province, at different vegetation stages by an Illumina Miseq sequence analysis of the 16S ribosomal ribonucleic acid and internal transcribed spacer genes. Significant shifts of the taxonomic composition of the bacteria and fungi were observed in the four stages. *Burkholderia*-*Caballeronia*-*Paraburkholderia* were the most dominant group in stage T1 and T2; the genera *Allorhizobium*-*Neorhizobium*-*Pararhizobium*-*Rhizobium* were decreased significantly from T1 to T2; and the genera *Acidothermus* and *Bacillus* were the most dominant in stage T3 and T4, respectively. Meanwhile, *Neocosmospora*, *Saitozyma*, *Penicillium*, and *Trichoderma* were the most dominant genera in the stage T1, T2, T3, and T4, respectively. Among the core microbiota, the dominant bacterial genera were *Burkholderia-Caballeronia-Paraburkholderia* and *Bacillus*, and the dominant fungal genera were *Saitozyma* and *Trichoderma*. In addition, we identified five bacterial genera and five fungal genera that reached significant levels during development. Finally, we constructed the OTU (top 30) interaction network of bacteria and fungi, revealed its interaction characteristics, and found that the bacterial OTUs exhibited more extensive interactions than the fungal OTUs. Understanding the rhizosphere soil microbial diversity characteristics of the areca palm could provide the basis for exploring microbial association and maintaining the areca palm’s health.

## 1. Introduction

The areca palm (*Areca catechu* L.), an important economic and medicinal crop widely cultivated in the tropical zone, has been utilised extensively in agriculture, industry, and for religious purposes [1,2]. The areca palm is the second-largest tropical cash crop, and is becoming one of the main economic pillars of Hainan, China. Most attention given to the areca palm has been dedicated to revealing its genetic variability [3], genome assembly [4], elucidation of its secondary metabolite pathways by transcriptome sequencing [5], and the microbiome and metabolome analysis of its leaves [6]. Despite the economic importance of the areca palm, little work has been done to explore its rhizosphere microbiome. Numerous cases on the plant root microbiome have demonstrated that microbes play key roles in supporting plant nutrition and health [7,8]. In return, the compounds released by the plants can also affect the rhizosphere soil directly, and the rhizosphere microorganisms are generally influenced by the soil type [7], environmental conditions [9], plant genotype [10], and plant developmental stage [11]. In its entirety, the rhizosphere is an environment where a large number of microbes communicate extensively, and the plant shapes the rhizosphere microbiome [8,12].

Results of the root exudation patterns of phytochemicals during plant development, together with community profiling and transcriptome analysis of their associated rhizosphere microbiota, indicated that the microbiota trigger certain beneficial functions of the plants through the exudation of particular chemical compounds that vary with the changing requirements during plant development [13]. Further, a subset of soil microbes, for the specific needs of the plants, can be recruited by the plant exudates [14]. These complex, plant-associated microbiota are also deemed the second genome of the plant, which is crucial for plant health [15]. Thus, plant–microbe interactions are of specific interest, not only to reveal their role during plant growth and development, but also to investigate their relationships to sustainable crop health.

Studies have demonstrated that the areca palm is a source of alkaloids, flavonoids, and carotene, and its active components are used to cure leukoderma, cough, worms, obesity, and nasal ulcers [16]. Can the root exudates affect the microbiota community of the rhizosphere? What are the core rhizosphere microbiome characteristics of the different growth stages of the areca palm? Taking into account the above, we aim to (i) characterize the rhizosphere soil microbiota diversity at the four different development stages of the areca palm, (ii) decipher the dynamic variation characteristics and the core microbial groups of the areca palm rhizosphere, and (iii) explore the interaction networks of bacteria and fungi. The results will give us a novel view of microbiota variation along with the areca palm’s growth, and will lay a foundation for further studying on the rhizosphere microorganisms keeping the areca palm healthy.

## 2. Materials and Methods

### 2.1. Soil Samples Collection and Deoxyribonucleic Acid (DNA) Extraction

**Figure 1:**
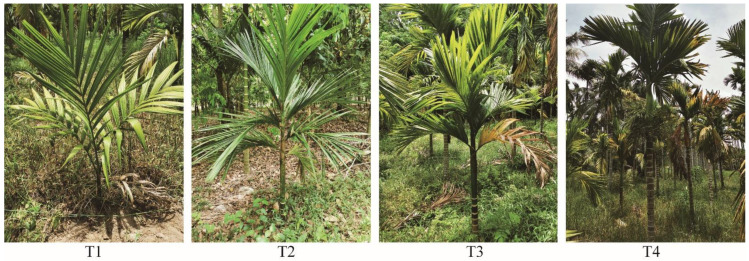
Areca palm trees of four different growth stages. T1 stage was about eight months old, T2 stage was about two years old, T3 stage was about three years old, and T4 stage was about seven years old.

Soil samples were collected (25 May 2020) from Longgun Town, Wanning County, Hainan Province, China, with due permission from the Wanning County government. The areca palm rhizosphere soil sampling sites were located at 18.39°21′73′′ N, 109.55°40′41″ E. The areca palm plants have been continuously planted for more than seven years at the above sampling sites. The areca palms were chosen at different growth stages (Figure 1), including the T1 (about eight months old, ≈1.0 m), T2 (about two years old, ≈1.8 m), T3 (about three to four years old, ≈3.0 m), and T4 stages (about seven years old, ≈5.0 m). First, the grass that covered the sample soil surface was cleared, the root soil was dug using a hoe, and then the roots coming in light (≈10 cm depth) were collected using scissors and put into a sterile plastic bag. The roots at three different locations of each selected tree were then collected and combined as one sample. At each growth stage (T1, T2, T3, T4), the roots from three healthy trees with similar growth conditions were chosen as the three repeats. Moreover, three soil samples of the areca palm were filled—with no areca palm trees planted—and were defined as the bulk soil. Similarly, the three bulk soil samples (at 10 cm depth with approximately 10 g per soil sample) were collected from the different sites. All the samples were stored in sterile plastic bags and immediately transported to the laboratory in an icebox. In the laboratory, the larger soil particles were removed from the roots first, then the roots were put in a sterile centrifuge tube (50 mL) with 20 mL of PBS buffer and shocked for 5 min in the Vortex oscillator. The roots were then taken out, and the tubes were centrifuged for 10 min at 12,000 rpm. The supernatant was removed and the sediment was collected. The collected sediment samples were stored at −20 °C until the 16S ribosomal deoxyribonucleic acid (rDNA) and internal transcribed spacer (ITS) sequencing and analysis.

Aliquots (0.25 g) of the soil samples were processed to extract the DNA using the PowerSoil^®^ kit (MOBIO, Carlsbad, CA, USA), which is based on bead beating, according to the manufacturer’s instructions. The extraction blanks were processed in parallel throughout the full procedure as negative controls to evaluate any potential DNA contamination from the reagents. The extracted DNA samples were analysed using a NanoDrop 2000 UV-visible spectrophotometer (Thermo Scientific, Wilmington, DE, USA). The DNA quality was confirmed using 1% agarose gel electrophoresis. The extracted DNA samples were selected and used to conduct a microbial community analysis through polymerase chain reactions (PCR) using 16S rDNA primers 338F: 5′-ACTCCTACGGGAGGCAGCAG-3′ and 806R: 5′-GGACTACHVGGGTWTCTAAT-3′ [17], as well as ITS primers (ITS1: 5′-CTTGGTCATTTAGAGGAAGTAA-3′ and ITS2R: 5′-GCTGCGTTCTTCATCGATGC-3′) [18]. The PCRs were performed as described by Li et al. [19]. The PCR products were further purified using the AxyPrep DNA Gel Extraction Kit (Axygen Biosciences, Union City, CA, USA), and the purified products were quantified using the QuantiFluor-ST (Promega, Madison, WI, USA). The above purified amplicons were then pooled in equimolar concentrations and paired-end sequenced (300 bp) using the Illumina MiSeq platform (Illumina, San Diego, CA, USA), according to the standard protocols of Shanghai’s Majorbio Bio-pharm Technology Co., Ltd. (Shanghai, China). Raw sequences were then trimmed for the barcode and primer sequences, any ambiguous bases, homopolymers > 6 bases, and filtered using the FASTX Toolkit 0.0.12 software to remove low-quality reads with Q values <20 and any bp less than 35 [20]. The paired-end reads were merged using the “fastq_mergepairs” command. High-quality sequences were then selected and dereplicated using the “fastq_filter” command and “derep_fulllength” command, respectively. The singletons were removed using the USEARCH-unoise3 algorithm and the chimeric sequences were removed using the “uchime_ref” command.

### 2.2. Diversity Analysis of Microbial Communities

The 16S rDNA sequences were amplified using 338F/806R primers and blasted in the Silva database (Release138 http://www.arb-silva.de (accessed on 15 July 2020). The ITS sequences were amplified using ITS1/ITS2 primers and blasted in the database for fungi (Unite, Release 8.0 http://unite.ut.ee/index.php (accessed on 15 July 2020). All sequences were analysed using the Majorbio I-Sanger online cloud platform (http://www.i-sanger.com (accessed on 15 July 2020). The related parameters and models used in the analysis have been listed as follows. The similarities and differences between samples were compared using the shared and unique OTUs of a Venn diagram, and the Student’s *t*-test was used to assess the significance level. Bar plot analyses were conducted at the genus level. The principal coordinates analysis (PCoA) of β-diversity was calculated based on the Bray–Curtis algorithm, the model of difference test between groups was an ANOSIM, and the number of replacements was 999. A Kruskal-Wallis H test and false discovery rate were used with a Scheffe cutoff value of 0.95 to compare the significance testing of the microbial community variance of soil samples at the genus level. The confidence interval (CI) method was used to calculate CI values. A network analysis of the top 30 OTUs of bacteria and fungi was conducted at the genus level to assess the correlation characteristics of the soil samples and the OTUs with thresholds of *r* ≥ 0.5 and *p* < 0.05, respectively. For these analyses, the Spearman correlation coefficient model was used with a cutoff of 0.5. The bacterial and fungal OTUs used in this study are listed in the Supplemental Information Tables S1 and S2, respectively.

## 3. Results and Discussion

### 3.1. Bacterial and Fungal Diversity of the Areca Palm Rhizosphere

**Figure 2:**
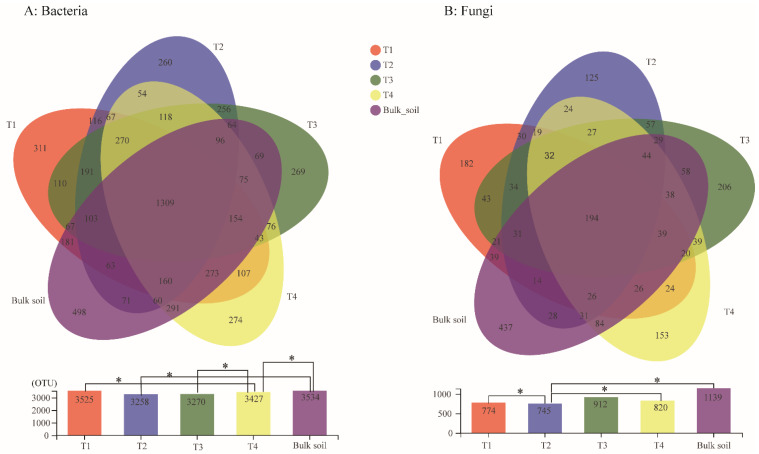
Venn analysis of shared and unique bacteria (**A**) and fungi (**B**) OTUs among four different growth stages and bulk soil samples. OTUs defined by 97% sequence similarity and Student’s *t*-test of OTU level among the soil samples were detected. * means *p* ≤ 0.05.

**Figure 3:**
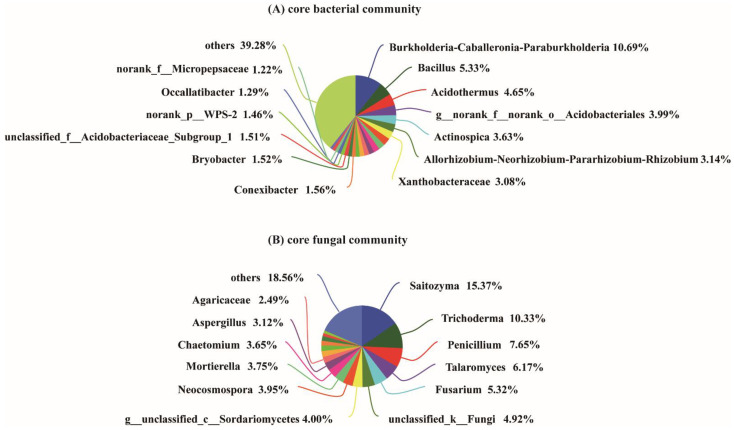
Core bacterial (**A**) and fungal (**B**) genera of four development stages of the areca palm. Core genera means that the genera existed in the rhizosphere soil of the four stages. “others” indicates genera with relative abundance less than 1%.

The bacterial diversity of the soil samples collected from the four different growth stages and the bulk soil was assessed using phylotype taxonomy. A total of 15 soil samples were sequenced and 715,276 raw reads were obtained. The results revealed a total of 6056 OTUs, which belonged to 913 genera and 1900 species. There were 3525, 3258, 3270, 3427, and 3534 OTUs in the T1, T2, T3, T4, and bulk soil samples, respectively. The number of core OTUs (found at all samples) was 1309, accounting for 21.62% of the total OTUs. The T1, T2, T3, and T4 stages exhibited 311, 260, 269, and 274 unique OTUs, respectively (Figure 2A). The result of the Student′s *t*-test further indicated that the Sobs index of the OTU level between T1 and T4, T2 and bulk soil, T3 and T4, and T4 and bulk soil were significant (Figure 2A). A total of 1579 core OTUs from 461 genera were detected in the samples T1, T2, T3, and T4, among which the dominant genera were *Burkholderia-Caballeronia-Paraburkholderia* (10.69%), *Bacillus* (5.33%), *Acidothermus* (4.65%), norank_o_Acidobacteriales (3.99%, belongs to Order Acidobacteriales), *Actinospica* (3.63%), *Allorhizobium-Neorhizobium-Pararhizobium-Rhizobium* (3.14%), and *Xanthobacteraceae* (3.08%) (Figure 3A).

In addition, for the analysis of the fungal OTUs, 15 soil samples were sequenced and 1,025,914 raw reads were obtained. A total of 2936 fungal OTUs belonging to 596 genera and 967 species were identified among the five sample groups. There were 774, 745, 912, 820, and 1139 fungal OTUs in the T1, T2, T3, T4, and bulk soil samples, respectively. The Venn diagram analysis demonstrated that the core fungal OTUs were 194, accounting for 6.61% of the total OTUs. The T1, T2, T3, and T4 stages exhibited 182, 125, 206, and 153 unique fungal OTUs, respectively (Figure 2A). Furthermore, the β-diversity analysis indicated that the OTUs between T1 and T2, T2 and T4, and T2 and bulk soil reached a significant level (Figure 2B). Among the samples T1, T2, T3 and T4, the 226 core fungal OTUs belonged to 130 genera, and the main genera were *Saitozyma* (15.37%), *Trichoderma* (10.33%), *Penicillium* (7.65%), *Talaromyces* (6.17%), *Fusarium* (5.32%), unclassified_k__Fungi (4.92%), g_unclassified_c_Sordariomycetes (4%), *Neocosmospora* (3.95%), *Mortierella* (3.75%), *Chaetomium* (3.65%), *Aspergillus* (3.12%), and *Agaricaceae* (2.49%) (Figure 3B).

### 3.2. Bacterial Composition at Different Growth Stages

**Figure 4:**
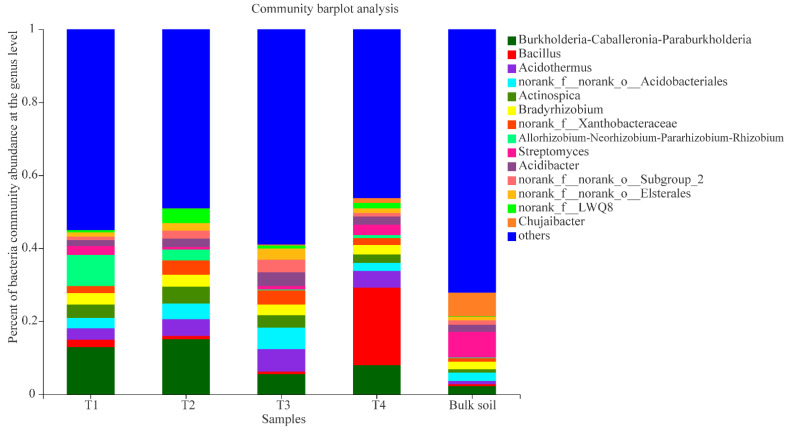
Composition of bacterial communities (T1, T2, T3, T4, and bulk soil) at the genus level; “others” indicates genera with relative abundance less than 0.03%.

The primary bacterial genera in the areca palm T1 rhizosphere samples were *Burkholderia*-*Caballeronia*-*Paraburkholderia* (relative abundance 12.91%), *Allorhizobium*-*Neorhizobium*-*Pararhizobium*-*Rhizobium* (8.47%), *Actinospica* (3.64%), *Bradyrhizobium* (3.15%), *Acidothermus* (3.09%), norank_f_norank_o_Acidobacteriales (2.86%), *Streptomyces* (2.51%), and *Bacillus* (2.06%); the genera *Burkholderia*-*Caballeronia*-*Paraburkholderia* (15.03%), *Acidothermus* (4.59%), norank_f_norank_o_Acidobacteriales (4.28%), *Actinospica* (4.66%), *Bradyrhizobium* (3.25%), norank_f_Xanthobacteraceae (3.93%), norank_f_LWQ8 (3.99%)*, Allorhizobium-Neorhizobium-Pararhizobium-Rhizobium* (2.96%), *Acidibacter* (2.32%), and norank_f_norank_o_Elsterales (2.14%) were the dominant groups in the T2 rhizosphere samples; the genera *Acidothermus* (6.05%), norank_f_norank_o_Acidobacteriales (5.94%), *Burkholderia*-*Caballeronia*-*Paraburkholderia* (5.57%), norank_f_Xanthobacteraceae (3.93%), *Acidibacter* (3.75%), *Actinospica* (3.39%), norank_f_norank_o_Subgroup_2 (3.45%), norank_f_norank_o_Elsterales (3.02%), and *Bradyrhizobium* (2.87%) were the dominant groups in the T3 rhizosphere samples; and the genera *Bacillus* (21.21%), *Burkholderia-Caballeronia-Paraburkholderia* (7.99%), *Acidothermus* (4.57%), norank_f_norank_o_Acidobacteriales (2.22%), *Streptomyces* (2.85%), *Bradyrhizobium* (2.61%), *Actinospica* (2.32%), and *Acidibacter* (2.22%) were the dominant groups in the T4 rhizosphere samples (Figure 4). In addition, the result of the PCoA also revealed that the bacterial genera of the four growth stages and bulk soil samples were varied distinctly, and the PC1 axis showed 45.2% variation among them (Figure S1).

### 3.3. Fungal Composition at Different Growth Stages

**Figure 5:**
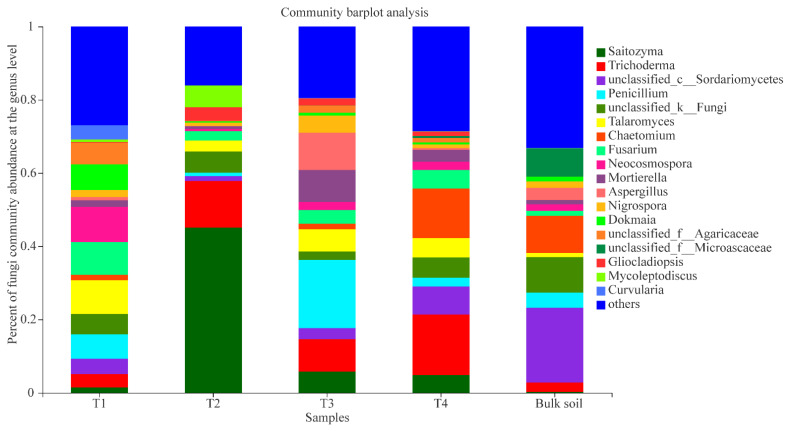
Composition of fungal communities (T1, T2, T3, T4, and bulk soil) at the genus level; “others” indicates genera with relative abundance less than 0.03%.

The fungal composition at the different growth stages and bulk soil samples were also analysed. In the stage T1, the primary fungal genera were *Neocosmospora* (9.60%), *Talaromyces* (9.25%), *Fusarium* (8.99%), *Penicillium* (6.57%), unclassified_f_Agaricaceae (5.88%), unclassified_k_Fungi (5.54%), unclassified_c_Sordariomycetes (4.21%), *Curvularia* (3.87%), and *Trichoderma* (3.65%); the dominant genera at stage T2 were *Saitozyma* (45.09%), *Trichoderma* (12.73%), *Mycoleptodiscus* (5.93%), unclassified_k_Fungi (5.79%), *Gliocladiopsis* (3.73%), *Talaromyces* (2.97%), and *Fusarium* (2.57%); at stage T3, the main groups were *Penicillium* (18.67%), *Aspergillus* (10.18%), *Trichoderma* (8.79%), *Mortierella* (8.77%), *Talaromyces* (6.08%), *Saitozyma* (5.76%), *Nigrospora* (4.70%), *Fusarium* (3.74%), unclassified_c_Sordariomycetes (3.07%), unclassified_k_Fungi (2.24%), and *Neocosmospora* (2.19%); and at stage T4, the dominant genera were *Trichoderma* (16.43%), *Chaetomium* (13.56%), unclassified_c_Sordariomycetes (7.70%), unclassified_k_Fungi (5.52%), *Talaromyces* (5.25%), *Fusarium* (5.08%), *Saitozyma* (4.87%), *Mortierella* (3.23%), *Penicillium* (2.41%), and *Neocosmospora* (2.29%) (Figure 5). The result of the PCoA analysis indicated that the fungal communities of the four growth stages and bulk soil samples were also dynamic, and the PC1 axis showed 21.87% variation among them (Figure S2).

### 3.4. Microbial Genera with Significant Differences among the Samples

**Figure 6:**
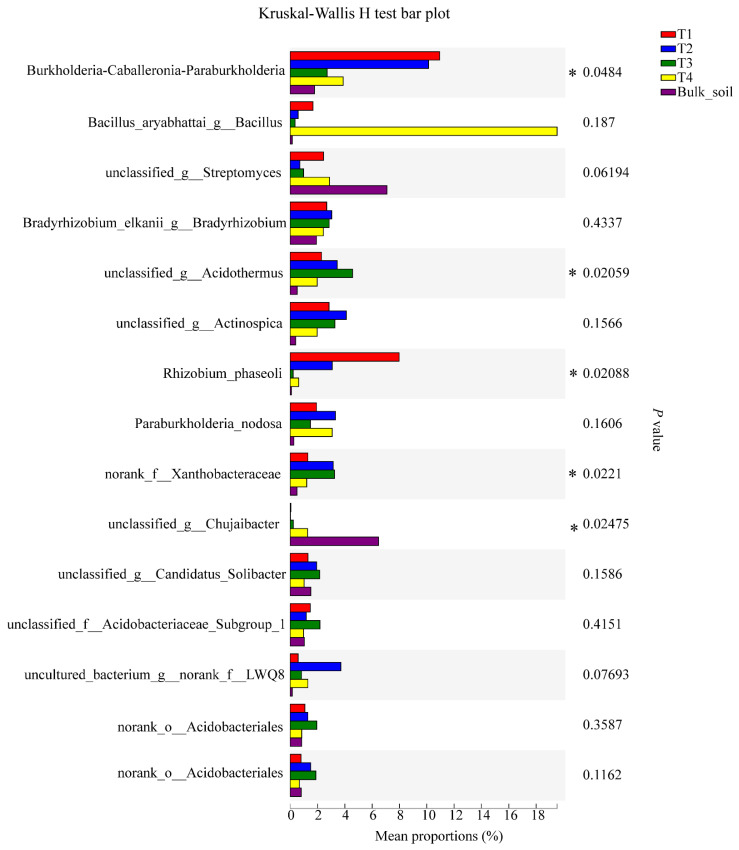
Significant differences in the abundance of bacterial genera among the soil samples, based on the Kruskal-Wallis H test (95% confidence interval, CI). * means *p* ≤ 0.05.

**Figure 7:**
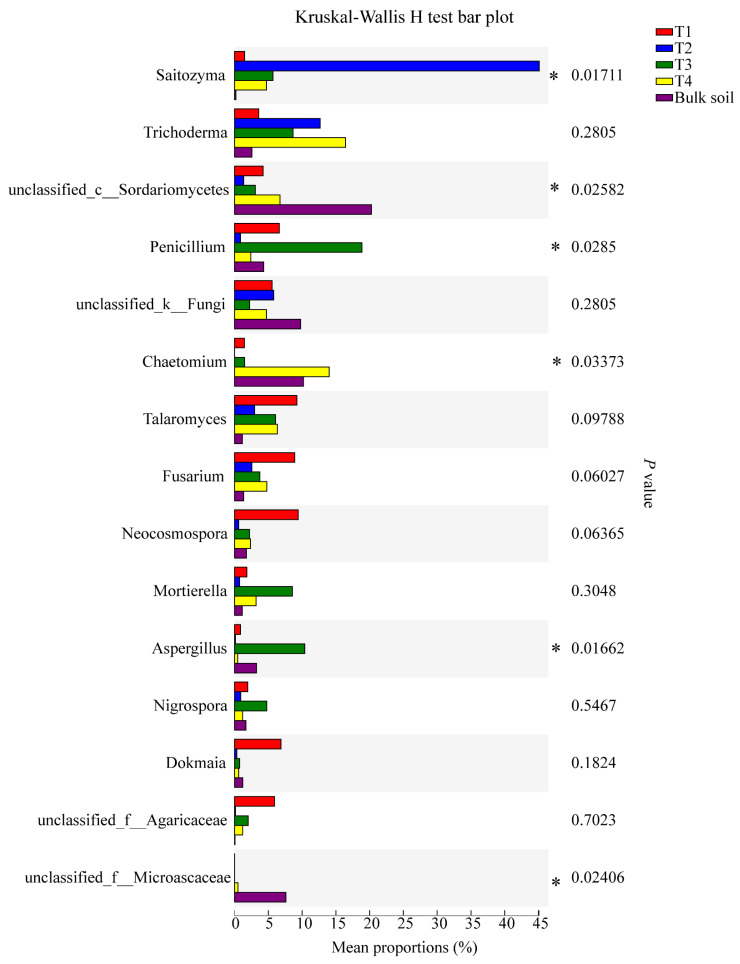
Significant differences in the abundance of fungal genera among the soil samples, based on the Kruskal-Wallis H test (95% confidence interval, CI). * means *p* ≤ 0.05.

For bacteria, the relative abundance of the genera *Burkholderia*-*Caballeronia*-*Paraburkholderia*, unclassified_g_Acidothermus, *Rhizobium phaseoli,* norank_f_Xanthobacteraceae, and unclassified_g_*Chujaibacter* reached significance (95% CI, *p* < 0.05) (Figure 6). For fungi, the relative abundance of the genera *Saitozyma*, *Penicillium*, *Chaetomium*, *Aspergillus*, and unclassified_f_Microascaceae were significant (95% CI, *p* < 0.05; Figure 7).

### 3.5. Bacterial and Fungal Network Analyses

**Figure 8:**
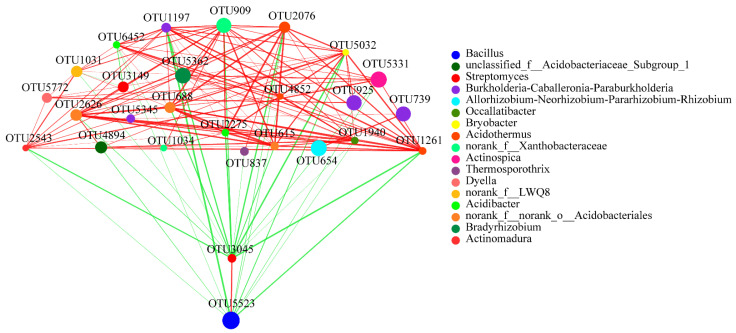
Bacterial interaction network analysis at the OTU level of four different growth stages. Different node colours represent different bacteria genera. Blue lines represent negative interactions; red lines represent positive interactions among different nodes. The OTUs with the thresholds of *r* ≥ 0.5 and *p* < 0.05 were selected.

We conducted a network analysis to reveal the symbiotic relationships of species in the areca palm soil samples. The network analysis of these abundant OTUs revealed the interaction between the rhizosphere soil bacteria and fungi. In the bacterial network, the results indicated extensive interactions among the identified genera. These abundant OTUs were from 17 genera, such as the main genera *Burkholderia*-*Caballeronia*-*Paraburkholderia* (four OTUs), the genus *Acidothermus* (three OTUs), the genus norank_f_norank_o_Acidobacteriales (two OTUs), *Streptomyces* (two OTUs), and norank_f_Xanthobacteraceae (two OTUs). Interestingly, OTU5523 (from *Bacillus*) only had a positive correlation with OTU3045 (from *Streptomyces*) and was negatively correlated with all the other OTUs. In addition, OTU5523 showed a strong negative correlation with OTU1197, while OTU3045 revealed a strong negative correlation with OTU2543, OTU5032 and OTU1261 (Figure 8).

**Figure 9:**
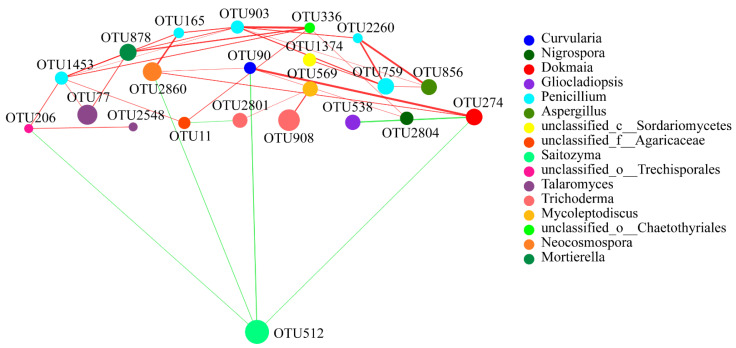
Fungal interaction network analysis at the OTU level of four different growth stages. Different node colours represent different bacteria genera. Blue lines represent negative interactions; red lines represent positive interactions among different nodes. The OTUs with the thresholds of *r* ≥ 0.5 and *p* < 0.05 were selected.

In the fungal network, results indicated that the relationship of the five OTUs (OTU165, OTU903, OTU759, OTU1453, and OTU2260) of *Penicillium* were positively correlated, and a strong correlation existed between OTU336 and OTU903; OTU512 had a high relative abundance and only showed a negative correlation with four OTUs (OTU90, OTU206, OTU274, and OTU2860), but a strong positive correlation was found between OTU90 and OTU274. A strong positive correlation also existed between OTU856 and OTU2260 (Figure 9).

## 4. Discussion

Rhizosphere soil is considered a highly complex and dynamic ecosystem. In this study, we compared the microbiota variation in the four typical growth stages of an areca palm under the same planting region. The results demonstrated that the bacterial and fungal communities changed distinctly during the development of the areca palm, for the core bacterial and fungal OTUs only accounted for 28.4% (1579/5558) and 13.2% (226/1717), respectively. Thus, the composition and diversity of the rhizosphere microbiota were dynamic during the development of the areca palm (Figure 2 and Figure 3), which is consistent with the study’s findings that the host genotype and age can contribute to the complexity of the microbiome assembly in natural environments [21,22]. For the moment, except for a report on the microbiome of the areca palm leaf [6], this study is the first report on the rhizosphere soil microbiome related to the different growth stages.

Based on the positive and negative interactions, these key OTUs subsequently help to construct a more stable rhizosphere soil microbiota with greater diversity, for numerous previous results have shown that soils with greater bacterial diversity facilitate nutrient cycling by the microorganisms, promote plant growth, and protect the plants from pathogens [23,24]. Our network analysis results of the bacteria and fungi showed that there is greater bacterial diversity and more complex interactions existed than in the fungal network (Figure 8 and Figure 9). In particular, the OTU3045 from *Streptomyces* and OTU5523 from *Bacillus* exhibited extensive negative interactions to other genera, but have a positive interaction between them, which may balance or limit the other microorganisms, and subsequently help to construct a more stable bacterial community with greater diversity. Notably, the abundance level of the *Bacillus* in T4 was absolutely higher than other stages of areca palm growth. Numerous studies have shown that *Bacillus* spp. are beneficial microorganisms that produce a vast array of biologically active molecules that inhibit pathogens [25]. For example, surfactin, iturin, and fengycin produced by *Bacillus* spp. have been applied to control the diseases caused by *Ralstonia solanacearum* [26], *Rhizoctonia solani* [27], *Pythium aphanidermatum* [28], and *Podosphaera fusca* [29]. Moreover, we also observed that the organisms from the genus *Bradyrhizobium* were stable in all soil samples. Several studies have established that *Bradyrhizobium* spp. play a critical role in nitrogen fixation and soil fertility, and the organisms from this genus are also used to evaluate the toxicity of pollutants [30]. Therefore, the genus *Bradyrhizobium* should also be key organisms in the areca palm rhizosphere.

Moreover, results of the fungal interaction network analysis also indicated that the OTU512 from *Saitozyma* showed a negative interaction with other genera (Figure 9). Together with the fact that *Saitozyma* was one of the dominant fungal genera of the T2, T3, and T4 stages—and it existed at stage T2 with a very high richness (Figure 5)—the species of *Saitozyma* should be considered the key fungi that exists in the areca palm, and might play a key role in balancing the stability of the fungal interaction network.

Based on the fungi diversity results, it was obvious that the fungal composition changed distinctly with the development of the areca palm, but the structure of the fungal nutrient types in each sample was highly similar among the four different growth stage soil samples, with saprophytes being the absolute dominant type. In summary, the dominant fungi in the areca palm rhizosphere sample mainly consisted of the Pathotroph-Saprotroph-Symbiotroph and Symbiotroph types, such as the genera *Fusarium*, *Talaromyces*, and *Saitozyma,* which were dominant—and found in all the soil samples. It is noteworthy that *Fusarium* spp. can result in yellow leaf disease of the areca palm, and its death in the field, which seriously affects the development of Hainan’s local agriculture and food processing industry [6]. It is a filamentous fungal genus containing many agronomically important plant pathogens and mycotoxin producers [31]. Although all the samples were from healthy plants, we detected a high relative abundance of *Fusarium* in these samples, which might cause yellow leaf disease once the environment is beneficial to *Fusarium* spp.

## 5. Conclusions

We profiled the structural variability of the rhizosphere microbiomes in field-grown areca palms across the development stages, which provide new insights into the rhizosphere microbiota variation and areca palm development. Importantly, our report demonstrated that both the diversity and composition of the rhizosphere microbiota changed significantly during plant development. Furthermore, *Burkholderia-Caballeronia-Paraburkholderia* were the most dominant bacterial genera of stage T1 and T2; *Acidothermus* and *Bacillus* were the most dominant bacterial genera of T3 and T4, respectively; and *Neocosmospora*, *Saitozyma*, *Penicillium*, *Trichoderma* were the most dominant genera of stage T1, T2, T3, and T4, respectively. Finally, the bacterial and fungal interaction network further indicated that there was more bacterial presence and more extensive interactions than were found in the fungi. These findings could provide the basis for more detailed studies of the identified key OTUs and gain further insight into the complex host–microbe interactions of the areca palm.

## Data Availability

Data available on request, the raw reads of 16S and ITS MiSeq data were deposited into the NCBI Sequence Read Archive database (accession No.: SAMN20691801).

## Supplementary Materials

The following are available online at https://www.mdpi.com/article/10.3390/plants10122706/s1. Figure S1: PCoA analysis of bacterial communities at the genus level in the soil samples. Figure S2: PCoA analysis of fungal communities at the genus level in the soil samples. Table S1: Bacterial OTUs used in this study. Table S2: Fungal OTUs used in this study.
