# Supplementary material for: Microbial Diversity Characteristics of Areca Palm Rhizosphere Soil at Different Growth Stages
Source: Plants (Basel). 2021 Dec 9;10(12):2706. doi: 10.3390/plants10122706 (PMC8705836; doi:10.3390/plants10122706)
Supplement: Supplementary file 1 [file plants-10-02706-s001.zip › plants-1362750-supplementary.pdf]

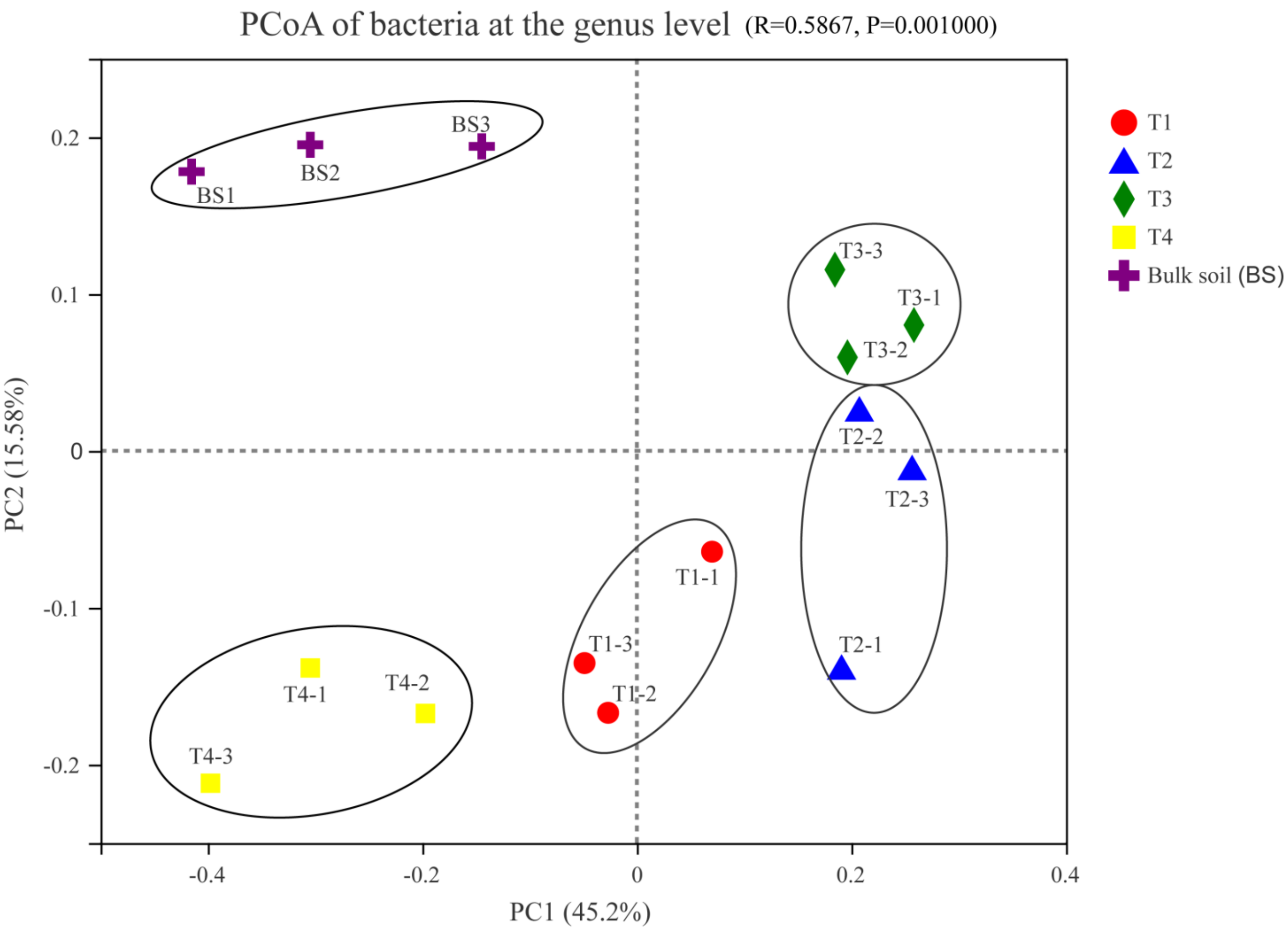

Figure S1. PCoA analysis of bacterial communities at the genus level in the soil samples.

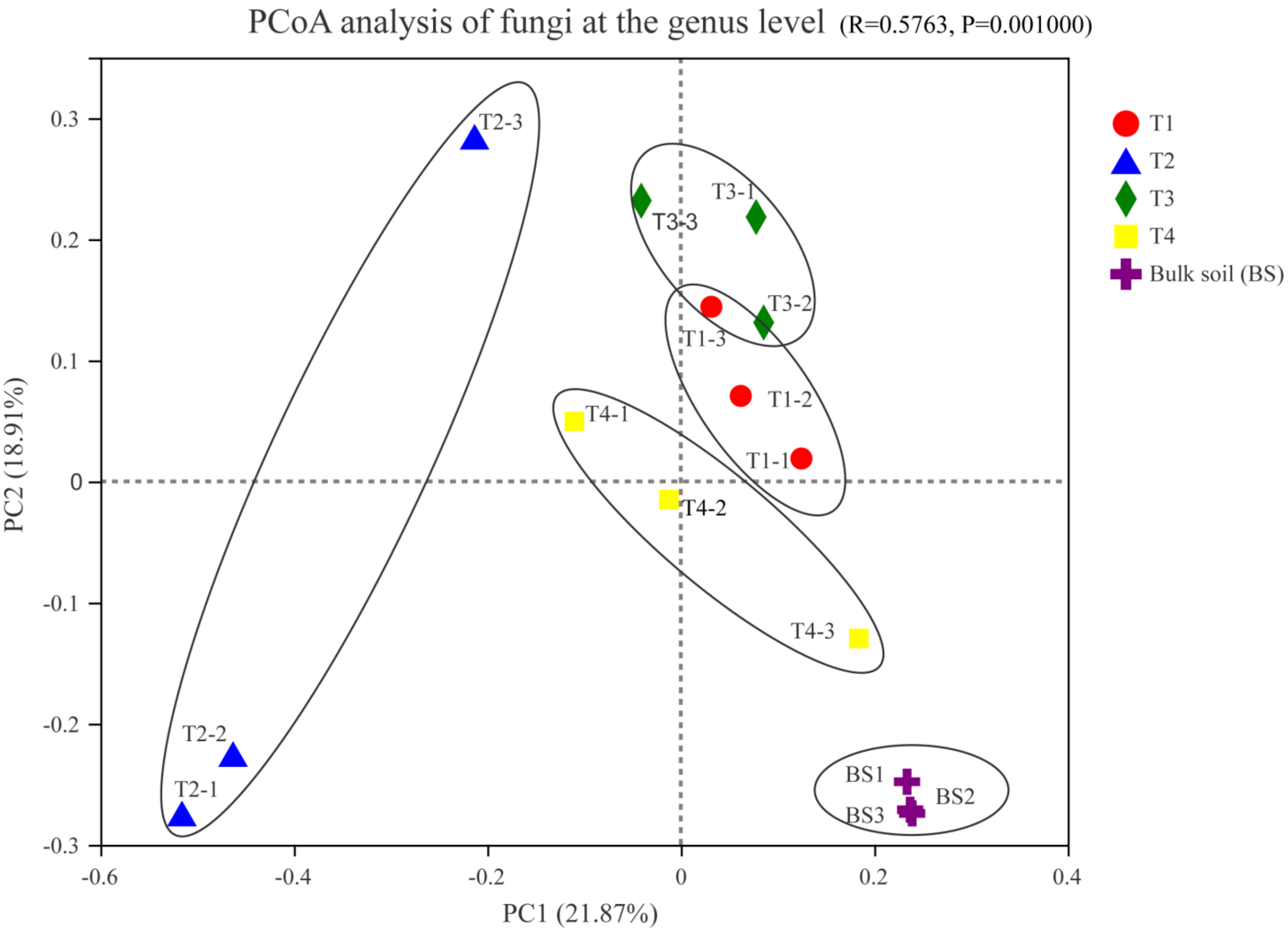

Figure S2. PCoA analysis of fungal communities at the genus level in the soil samples.
